# Giant Magneto‐Optical Effects in Two‐Dimensional Flat‐Band Antiferromagnets

**DOI:** 10.1002/advs.77173

**Published:** 2026-08-31

**Authors:** Ping Yang, Wanxiang Feng, Siyuan Liu, Shan Guan, Liwei Wen, Wei Jiang, Gui‐Bin Liu, Yugui Yao

**Affiliations:** ^1^ Key Lab of Advanced Optoelectronic Quantum Architecture and Measurement (MOE), Beijing Key Laboratory of Quantum Matter State Control and Ultra‐Precision Measurement Technology, and School of Physics Beijing Institute of Technology Beijing China; ^2^ Department of Physics Tianjin Renai College Tianjin China; ^3^ State Key Laboratory of Semiconductor Physics and Chip Technologies Institute of Semiconductors Chinese Academy of Sciences Beijing China; ^4^ College of Science Henan University of Engineering Zhengzhou China; ^5^ International Center for Quantum Materials Beijing Institute of Technology Zhuhai China

**Keywords:** antiferromagnets, flat‐band, magneto‐optical

## Abstract

In this work, we reveal giant magneto‐optical responses in two‐dimensional (2D) antiferromagnets with nearly flat electronic bands, based on first‐principles calculations and group‐theoretical analysis. We identify a record‐large second‐order magneto‐optical Schäfer–Hubert (SH) effect—featuring a polarization rotation angle of $28^{\circ }$—in monolayer antiferromagnetic ${\mathrm{RuOCl}}_{2}$, driven by flat‐band–enhanced interband optical transitions. Both the valence and conduction bands exhibit pronounced directional flatness, giving rise to highly anisotropic optical absorption and broadband hyperbolic frequency windows spanning the entire visible spectrum. This anisotropy leads to an exceptionally strong linear dichroism (LD) reaching 50%, far exceeding values reported in other 2D magnetic systems. Remarkably, the giant SH effect and LD appear at distinct photon energies, reflecting a momentum–direction–dependent crossover between flat and dispersive bands. Both responses are further amplified with increasing ${\mathrm{RuOCl}}_{2}$ film thickness. Our results establish flat‐band antiferromagnets as a fertile platform for realizing giant nonlinear magneto‐optical effects and open new avenues for 2D opto‐spintronic device applications.

## Graphical abstract

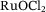
, a two‐dimensional antiferromagnet, hosts energy bands with pronounced directional flatness. This study demonstrates that the flat‐band‐induced optical transitions yield strongly anisotropic absorption and a record‐large magneto‐optical Schafer–Hubert effect, alongside exceptional linear dichroism exceeding 50%. These giant effects persist and are further enhanced in few‐layer films, establishing a novel material platform for directionally selective opto‐spintronic devices.

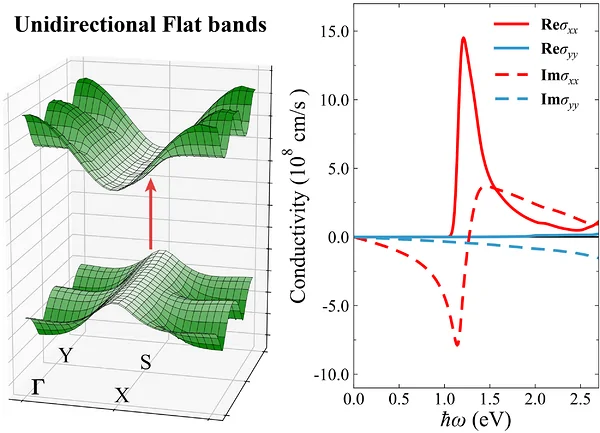

## 1 Introduction

Antiferromagnets, with vanishing net magnetization, terahertz‐scale spin dynamics, and inherent robustness to external perturbations, provide an appealing platform for high‐density, high‐speed, and secure spintronic applications [1, 2, 3, 4]. Magneto‐optical techniques offer a contactless, time‐resolved probe of spin dynamics [5, 6, 7, 8], and enable ultrafast control of spin configurations via all‐optical switching and optically driven spin currents [9]. However, in conventional collinear antiferromagnets, linear magneto‐optical responses—such as the Kerr and Faraday effects—are typically suppressed due to symmetry constraints arising from combined PT or Tτ operations, where P, T, and τ denote spatial inversion, time reversal, and fractional translation, respectively [10, 11]. Circumventing these constraints requires accessing higher‐order optical processes. Nonlinear effects such as second harmonic generation, which scales with the square of the electric field, and the Schäfer–Hubert (SH) [12] and Voigt [13] effects, which are quadratic in magnetization, provide viable alternatives. While second harmonic generation is forbidden in centrosymmetric systems, the SH and Voigt effects remain symmetry‐allowed and directly probe antiferromagnetic (AFM) order. Nonetheless, their typically weak signals pose a major challenge for experimental observation.

Flat‐band systems, characterized by nearly dispersionless energy–momentum relations, host electronic states with vanishing group velocity and strong spatial localization. These features enhance Coulomb interactions, stabilizing a rich landscape of correlated phases, including ferromagnetism [14, 15, 16, 17], superconductivity [18, 19], Wigner crystallization [20, 21], and fractional Chern insulators [22, 23]. Their intrinsic tendency to promote magnetic order and enhance interband transitions makes flat bands a natural setting for exploring interaction‐driven magneto‐optical phenomena. Specifically, the large joint density of states and enhanced transition matrix elements in flat‐band systems are expected to boost both linear and nonlinear magneto‐optical responses. Despite this promise, magneto‐optical effects in flat‐band materials remain largely unexplored. Prior studies have primarily focused on linear responses in idealized lattice models, such as the α‐$T_{3}$ model [24, 25], Kane model [26, 27], square lattices [28], and twisted bilayers [29, 30]. In contrast, second‐order magneto‐optical effects—such as the SH effect—in realistic flat‐band AFMs remain virtually unaddressed.

In this work, we employ first‐principles calculations combined with group‐theoretical analysis to demonstrate that monolayer AFM ${\mathrm{RuOCl}}_{2}$ exhibits a record‐large second‐order magneto‐optical SH effect, featuring a polarization rotation angle of $28^{\circ }$. This giant response originates from resonant interband optical transitions involving nearly flat bands. We further find that both the valence and conduction bands exhibit pronounced directional flatness, leading to strongly anisotropic optical absorption along orthogonal crystallographic axes and giving rise to broadband hyperbolic frequency windows spanning the entire visible spectrum. By analyzing dipole selection rules and band topology, we identify the microscopic origin of the anisotropic transitions. This optical anisotropy results in giant linear dichroism (LD) reaching 50%, surpassing all previously reported values in two‐dimensional (2D) magnetic materials. Notably, the SH effect and LD emerge at distinct photon energies, reflecting a momentum–direction‐dependent crossover between flat and dispersive bands. Finally, we show that both the SH effect and LD are significantly enhanced with increasing ${\mathrm{RuOCl}}_{2}$ film thickness. These findings establish a direct link between flat‐band physics and large anisotropic magneto‐optical responses, opening new avenues for 2D opto‐spintronic device applications.

## 2 Results and Discussion

**FIGURE 1:**
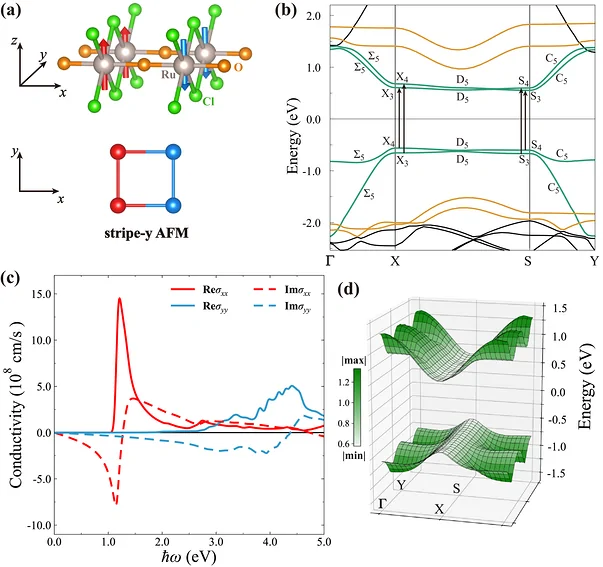
(a) Crystal and magnetic structures of monolayer ${\mathrm{RuOCl}}_{2}$ with the stripe‐y AFM configuration. In the top view, red and blue spheres represent Ru atoms with spin‐up and spin‐down orientations, respectively; nonmagnetic atoms (O and Cl) are omitted for clarity. (b) Relativistic band structure of monolayer stripe‐y AFM ${\mathrm{RuOCl}}_{2}$, with irreducible co‐representations indicated near the band edges. Two groups of bands—exhibiting nearly flat and strongly dispersive behavior along orthogonal crystallographic directions (Γ–X/S–Y and X–S)—are highlighted in green and gold, respectively. Vertical solid black arrows denote symmetry‐allowed optical transitions induced by x‐ and y‐polarized light at the X and S points. (c) Optical conductivity of monolayer stripe‐y AFM ${\mathrm{RuOCl}}_{2}$. (d) Two‐dimensional band structures of the topmost valence and lowest conduction bands. The color map represents the energy difference relative to the band‐edge extrema.

Bulk ${\mathrm{RuOCl}}_{2}$ crystallizes in an orthorhombic layered structure with the crystallographic space group Immm (No. 71) [31]. In the monolayer form, Ru atoms are connected via a single in‐plane O atom along the x direction and via two Cl atoms—positioned above and below the Ru plane—along the y direction (Figure 1a). Several magnetic configurations of monolayer ${\mathrm{RuOCl}}_{2}$ are considered, including the ferromagnetic (FM) and three types of AFM states: stagger, stripe‐x, and stripe‐y (see Figure S1). Total energy calculations reveal that the stripe‐y AFM configuration is the most energetically favorable ground state (Table S1), which is in good agreement with previous theoretical results [32]. The magnetic space group and the corresponding magnetic point group of the stripe‐y AFM state belong to $P_{a}mma$ (No. 51.298) and $mmm.1^{\prime }$ (No. 8.2.25), respectively. The interlayer binding energy is calculated to be 14.64 meV/Å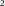 (Figure S2a), which lies within the typical range of 2D layered transition metal dichalcogenides (10–17 meV/Å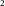) [33]. This relatively low binding energy suggests that monolayer ${\mathrm{RuOCl}}_{2}$ can be readily exfoliated from its bulk counterpart. Furthermore, the absence of imaginary frequencies in the phonon spectrum confirms the dynamic stability of the monolayer structure (Figure S2b).

After identifying the magnetic ground state, we proceed to investigate the electronic properties. As shown in Figure 1b, monolayer stripe‐y AFM ${\mathrm{RuOCl}}_{2}$ is a semiconductor with an indirect band gap of 1.11 eV. The valence (conduction) band extremum at the X point is 40 (14) meV higher in energy than that at the S point. Owing to the PT symmetry within the group $mmm.1^{\prime }$, all bands are doubly degenerate. The most remarkable feature of the band structure is its pronounced anisotropy. Near the band edges, along the X–S path, the top two valence bands and the bottom two conduction bands are nearly flat, whereas along the Γ–X and S–Y paths, the bands are highly dispersive. This indicates that charge carriers near the band edges exhibit significantly higher mobility along the Ru–O chains (aligned with the x direction) than along the Ru–Cl chains (aligned with the y direction). Interestingly, at energies further away from the band edges—specifically involving the third and fourth valence and conduction bands—the flat and dispersive characteristics are exchanged between the x and y directions. This implies a corresponding reversal of charge carrier mobility between the two orthogonal crystallographic directions.

This strong anisotropy in the electronic structure is reflected in the optical conductivity, which is also highly directional. Due to PT symmetry, the off‐diagonal components of the optical conductivity vanish, as $\mathrm{PT}\sigma_{xy}=-\sigma_{xy}$, leads to $\sigma_{xy}=0$ [10]. This implies that only the diagonal components need to be considered, as shown in Figure 1c. The absorptive parts of the optical conductivity—namely, the real parts of $\sigma_{xx}$ and $\sigma_{yy}$—originate from interband transitions. Notably, $\text{Re}\sigma_{xx}$ rises sharply just above the optical gap of 1.11 eV, reaching a pronounced peak at 1.2 eV, while $\text{Re}\sigma_{yy}$ remains negligibly small over the same energy range. According to the Kramers–Kronig relations, the imaginary part of $\sigma_{xx}$ exhibits a deep valley and a broad peak on either side of the $\text{Re}\sigma_{xx}$ peak at 1.2 eV. At photon energies above approximately 3.0 eV, the anisotropy in conductivity reverses, with both the real and imaginary parts of $\sigma_{yy}$ becoming larger than those of $\sigma_{xx}$.

Another remarkable feature of the optical conductivity is that the imaginary parts along the x and y directions have opposite signs, which corresponds to the permittivity condition $\text{Re}\varepsilon_{xx}\cdot \text{Re}\varepsilon_{yy}<0$ for hyperbolic dispersion. The predicted hyperbolic window of AFM ${\mathrm{RuOCl}}_{2}$ (1.3–4.4 eV and above 4.6 eV) fully covers the visible‐light range. Compared with the nonmagnetic ${\mathrm{RuOCl}}_{2}$, which exhibits a hyperbolic window of 0.08–3.36 eV [34], the AFM state shows a hyperbolic window of comparable width but at a higher frequency range. Although wide hyperbolic windows have been observed in various vdW crystals, such as ${\mathrm{Bi}}_{2}$ ${\mathrm{Se}}_{3}$ and ${\mathrm{Bi}}_{2}$ ${\mathrm{Te}}_{3}$ [35] (Figure S3), the majority of these kind of materials are uniaxial crystals. Their strong anisotropy arises from the radical contrast between in‐plane and out‐of‐plane bonding characters, and their optical axes lie along the out‐of‐plane direction, making this anisotropy difficult to harness practically for polarization optics [36]. In contrast, ${\mathrm{RuOCl}}_{2}$ is a biaxial crystal featuring strong in‐plane anisotropy. The hyperbolic window we predicted exceeds the experimentally measured values of other similar materials (Figure S3). Moreover, the imaginary parts of the permittivity, which represent dissipation, are quite small for some part of this hyperbolic window. These characters highlighting monolayer ${\mathrm{RuOCl}}_{2}$ as a promising 2D hyperbolic material.

To understand the sharp contrast between $\text{Re}\sigma_{xx}$ and $\text{Re}\sigma_{yy}$ around 1.2 eV, we first investigate whether the *y*‐polarized component is symmetry‐forbidden during band‐edge transitions. Using the MSGCorep package [37, 38], the calculated irreducible co‐representations at the X(S) point are $X_{3}$($S_{3}$) and $X_{4}$($S_{4}$), respectively, as labeled in Figure 1b. According to the dipole selection rules (see Figure S4 and relevant discussion in Supporting Information), the transitions are symmetry‐allowed for incident light polarized along both the x and y directions. Along the high‐symmetry paths Γ–X, X–S, and S–Y, only one irreducible co‐representation exists—$\Sigma_{5}$, $D_{5}$, and $C_{5}$, respectively—indicating that all interband transitions along these directions are symmetry‐allowed.

Next, we investigate whether the anisotropy of the optical conductivity is related to the dispersion characteristics of the valence band maximum (VBM). The longtidunal effective mass of the nth band can be written as [39]

(1) $$\frac{1}{m_{ii}^{*(n)}}=\frac{1}{m_{e}}+\sum_{m\neq n}\frac{2|\langle \psi_{n,k}|{\hat{v}}_{i}|\psi_{m,k}{\rangle |}^{2}}{E_{n,k}-E_{m,k}},$$

where $m_{e}$ is the mass of a free electron. The squared modulus of the velocity matrix element is always non‐negative. For electrons at the VBM, contributions from lower energy levels (m<n) to the effective mass are positive, while contributions from conduction bands (m>n) are negative. As the effective mass is typically negative, the contribution from the conduction band outweighs that from the lower valence bands, especially the states near the conduction band minimum (CBM), owing to their smaller denominator and hence larger weight. If the effective mass is small, then the velocity matrix element between the VBM and some bands near the CBM is likely to be large, and correspondingly, the band‐edge transitions can contribute a high optical conductivity. And conversely, a large effective mass corresponds to a low optical conductivity. This behavior is consistent with experimental and theoretical studies on black phosphorus [40, 41]. We propose that this behavior is applicable to the optical response induced by band‐edge transition in any direct bandgap semiconductor.

As shown in the 2D band structures of the top valence and bottom conduction bands (Figure 1d), the bands remain flat not only along X–S but also across a broad surrounding region, leading to persistently low optical absorption along the y direction from 1.0 to 3.0 eV. Thus, the strong absorption anisotropy ($\text{Re}\sigma_{xx}\gg \text{Re}\sigma_{yy}$) stems from the contrast between dispersive and flat bands along orthogonal directions. Notably, the absorption anisotropy reverses at higher photon energies (3.0–5.0 eV), with $\text{Re}\sigma_{xx}\ll \text{Re}\sigma_{yy}$. This stems from a shift in band character: the third and fourth valence and conduction bands become flat along Γ–X and S–Y, while bands along X–S grow more dispersive. This redistribution of flat and dispersive features across directions drives the reversal in absorption anisotropy.

Building on the anisotropic optical conductivity, we now discuss the resulting giant LD. The reflective LD, quantifying the relative intensity difference between orthogonally polarized lights, is defined as

(2) $$\text{LD}=\frac{R_{x}-R_{y}}{R_{x}+R_{y}},$$

where $R_{x(y)}={\left|r_{x(y)}\right|}^{2}$ is the reflectivity, and $r_{x(y)}$ is the complex field reflectivity at the sample surface, given by $r_{x(y)}=\left(1-{\tilde{n}}_{x(y)}\right)/\left(1+{\tilde{n}}_{x(y)}\right)$, with ${\tilde{n}}_{x(y)}$ being the effective refractive index of the thin film on an optically isotropic, nonmagnetic substrate [11]

(3) $${\tilde{n}}_{x(y)}=\frac{1-r_{x(y)}^{\prime }\beta_{x(y)}}{1+r_{x(y)}^{\prime }\beta_{x(y)}}n_{x(y)}.$$

Here, $\beta_{x(y)}=\exp\left(2i\omega d_{\text{eff}}n_{x(y)}/c\right)$, with ω being the light frequency, c the speed of light in vacuum, and $d_{\text{eff}}$ the effective thickness of the thin film. The interface reflectivity is $r_{x(y)}^{\prime }=\left(n_{x(y)}-n_{s}\right)/\left(n_{x(y)}+n_{s}\right)$, with $n_{x(y)}$ and $n_{s}$ the refractive indices of the film and substrate, respectively. The film's refractive index relates to its optical conductivity via $n_{x(y)}^{2}=1+\frac{4\pi i}{\omega }\sigma_{xx(yy)}$. In our calculations, the $n_{s}$ is obtained from optical constants databases (https://refractiveindex.info/), and the optical conductivities of monolayer (1L), bilayer (2L), and trilayer (3L) ${\mathrm{RuOCl}}_{2}$ were explicitly evaluated. As the thickness increases to up to 10 layers, the unidirectional flat bands persist, and their bandgap remains nearly the same as that of the bulk phase, as shown in Figure S5. Therefore, the optical response arising from band‐edge absorption is largely independent of layer thickness for samples of several nanometers thickness. We thus use the optical conductivity of the bulk phase to calculate the samples from 10 to 40 layers.

**FIGURE 2:**
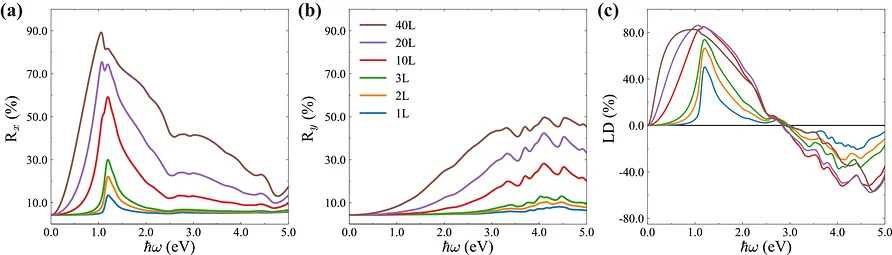
(a,b) Light reflectivity spectra along orthogonal crystallographic directions (x and y) directions and (c) linear dichroism spectra of ${\mathrm{RuOCl}}_{2}$ thin films with different numbers of layers.

The calculated reflectivity and LD spectra for ${\mathrm{RuOCl}}_{2}$ thin films (from monolayer to 40 layers) on a ${\mathrm{SiO}}_{2}$ substrate are shown in Figure 2. For the monolayer case, the reflectivity for x‐polarized light ($R_{x}$) exhibits a sharp peak of 14% at 1.2 eV, whereas the reflectivity for y‐polarized light ($R_{y}$) is nearly zero, remaining below 5% in the energy range of 3.0–5.0 eV. This behavior can be understood from the effective refractive index (Equation 3), which, for sufficiently thin films, can be approximated as

(4) $${\tilde{n}}_{x(y)}=n_{s}-\frac{i\omega d_{\text{eff}}}{c}\left(n_{x(y)}^{2}-n_{s}^{2}\right).$$

Around 1.2 eV, due to the pronounced anisotropy in optical conductivity ($\sigma_{xx}\gg \sigma_{yy}$) (Figure 1c), a strongly anisotropic refractive index arises, namely $n_{x}\gg n_{y}$. Given the negligibly small $n_{y}$, the corresponding effective refractive index ${\tilde{n}}_{y}$ approaches that of the substrate, ${\tilde{n}}_{y}\approx n_{s}$, as the second term in Equation (4) becomes negligible at low photon energies and in the monolayer limit. In contrast, the large $n_{x}$ leads to a significantly larger ${\tilde{n}}_{x}$, despite the small values of ω and $d_{\text{eff}}$. Consequently, the effective refractive index is also strongly anisotropic, ${\tilde{n}}_{x}\gg {\tilde{n}}_{y}$, resulting in a pronounced peak in $R_{x}$ and a negligible $R_{y}$ around 1.2 eV. This large difference between $R_{x}$ and $R_{y}$ produces a remarkable LD of 50.4% at 1.2 eV, as shown in Figure 2c. As the photon energy increases above 3.0 eV, the situation reverses, with $R_{x}<R_{y}$, which can be attributed to the reversal of optical conductivity anisotropy ($\sigma_{xx}\ll \sigma_{yy}$) (Figure 1c). Particularly, at 4.4 eV, the LD reaches as much as –20.1%. Benefiting from this energy‐dependent strong LD, the crystal orientation of a monolayer can be readily identified using azimuth‐dependent reflectance difference microscopy [42].

The reflectivity is monotonically enhanced by increasing the film thickness, while the LD presents a slightly complicated dependence on the film thickness. As the thickness increases from monolayer to 10 layers, $R_{x}$ grows much faster than $R_{y}$, particularly in the low‐energy range, below 3.0 eV (Figure 2a,b). As a result, the LD not only reaches a maximum of 84.7% at 1.2 eV but is also significantly enhanced across the visible light (e.g., 1.6–2.5 eV) and near‐infrared (0.4–0.9 eV) regions (Figure 2c). With further increases in thickness from 20 to 40 layers, the peak value of LD remains unchanged, but the LD stays relatively large across a broad energy range, particularly extending into the far‐infrared region below 0.4 eV. The thickness dependence of LD is also evident in the high‐energy range, specifically between 3.0 and 5.0 eV, where the LD reaches up to −57.6% at 4.7 eV (Figure 2c). The calculated LD in ${\mathrm{RuOCl}}_{2}$ reaches a record‐high value, surpassing those experimentally observed in all other known 2D materials (Table S2).

**FIGURE 3:**
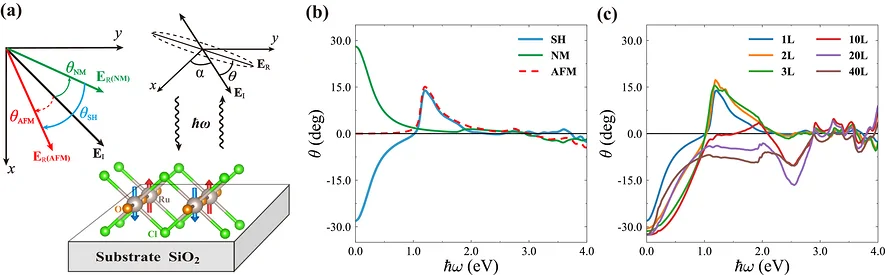
(a) Schematic illustration of the elliptically polarized light reflected from monolayer antiferromagnetic ${\mathrm{RuOCl}}_{2}$ on a ${\mathrm{SiO}}_{2}$ substrate. $E_{I}$ and $E_{R}$ denote the electric fields of the incident and reflected light, respectively. α is the angle between $E_{I}$ and the x‐axis, while θ represents the rotation angle of the polarization plane of the reflected light relative to the incident light. Specifically, $E_{\text{R(NM)}}$ and $E_{\text{R(AFM)}}$ correspond to the electric fields of the reflected light from NM and AFM ${\mathrm{RuOCl}}_{2}$, respectively. The associated rotation angles are $\theta_{\text{NM}}$ and $\theta_{\text{AFM}}$, and the magneto‐optical SH rotation angle is defined as $\theta_{\text{SH}}=\theta_{\text{AFM}}-\theta_{\text{NM}}$. (b) Rotation angle of monolayer NM and AFM ${\mathrm{RuOCl}}_{2}$, along with the corresponding magneto‐optical SH response. (c) SH rotation angle of ${\mathrm{RuOCl}}_{2}$ thin films with different numbers of layers.

Along with the strong LD, the reflected light can become elliptically polarized, accompanied by a rotation of the polarization plane relative to the incident light, as depicted in Figure 3a. When the electric field of the incident light ($E_{I}$) is oriented at an angle $\alpha =45^{\circ }$ with respect to the x‐axis, both the rotation angle (θ) and the ellipticity (ψ) reach their maximum values, given by [11]:

(5) θ=π4−12atan2Reχ1−|χ|2,

(6) ψ=12asin2Imχ1+|χ|2,

where $\chi =r_{y}/r_{x}$. A positive (negative) θ corresponds to a clockwise (counterclockwise) rotation, and a positive (negative) ψ indicates left‐handed (right‐handed) elliptical polarization. The rotation of the polarization plane and the corresponding ellipticity induced by magnetic order are referred to as the magneto‐optical SH effect, whereas the contributions arising from crystal anisotropy are termed natural linear birefringence (NLB). In stripe‐y AFM ${\mathrm{RuOCl}}_{2}$, due to unequal in‐plane lattice constants (Table S1), both the SH effect and NLB coexist. The NLB contribution can be evaluated in ${\mathrm{RuOCl}}_{2}$ by artificially removing its magnetic order, that is, by considering the system in a nonmagnetic (NM) state. Subsequently, the SH rotation angle is obtained by subtracting the NLB contribution from the results of AFM state [10]

(7) $$\theta_{\text{SH}}=\theta_{\text{AFM}}-\theta_{\text{NM}}.$$

In practice, $\theta_{\mathrm{AFM}}$ can be measured at low temperature in the magnetically ordered phase, while $\theta_{\mathrm{NM}}$ can be obtained at high temperature above the critical point where long‐range magnetic order vanishes. Their difference then defines the SH angle $\theta_{\mathrm{SH}}$.

The SH rotation angle of a monolayer ${\mathrm{RuOCl}}_{2}$ on a ${\mathrm{SiO}}_{2}$ substrate is shown in Figure 3b. In the AFM case, the rotation angle exhibits a frequency dependence similar to that of the LD spectrum, reaching a maximum of 15.1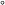 at 1.2 eV. This behavior essentially originates from the anisotropy in the optical conductivity. As derived in our previous work [11], the rotation of polarization plane is related to the difference in optical conductivity between the x and y directions, following the relation $\theta +i\psi \propto |\sigma_{xx}-\sigma_{yy}|$. The large difference in the real parts of $\sigma_{xx}$ and $\sigma_{yy}$ near 1.2 eV gives rise to the pronounced peak in $\theta_{\text{AFM}}$. In the NM case, the rotation angle $\theta_{\text{NM}}$ exhibits a sharp peak of 28.0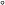 close to the zero‐frequency limit. This is attributed to the metallic nature of monolayer NM ${\mathrm{RuOCl}}_{2}$, which shows strong anisotropy in the intraband contributions to the reflectivity and LD below 0.5 eV (Figure S6). Given that the SH angle is defined as the difference between the rotation angles in AFM and NM states (Equation 7), and that $\theta_{\text{AFM}}$ is nearly zero below 1.0 eV, the SH angle in this low‐energy regime is dominated by and opposite in sign to $\theta_{\text{NM}}$. Conversely, for photon energies above 1.0 eV, the optical response in the NM state becomes nearly isotropic, and the SH angle closely follows $\theta_{\text{AFM}}$. Notably, the computed SH angle in ${\mathrm{RuOCl}}_{2}$ is several orders of magnitude larger than those observed in typical 2D and bulk magnetic materials (Table S2). Figure 3c shows the dependence of SH rotation angles on the thickness of ${\mathrm{RuOCl}}_{2}$ thin films. For films thinner than three layers, the SH angle profile resembles that of the monolayer, with the exception of a gradual enhancement in the 0–1.0 eV energy range. Starting from ten layers, the peak around 1.2 eV vanishes, and a new peak emerges, centered at 2.5 eV.

Finally, we discuss the broader relevance and generality of our findings. The stagger AFM state has an energy comparable to that of the stripe‐y AFM state (Table S1). Nevertheless, the stagger AFM phase of ${\mathrm{RuOCl}}_{2}$ also exhibits unidirectional flat bands near the Fermi level, which give rise to strong anisotropic conductivity, LD response, and SH angles (Figure S7). These characteristics are nearly identical to those of the stripe‐y AFM state, indicating that our conclusions for stripe‐y AFM ${\mathrm{RuOCl}}_{2}$ can be robustly extended to other AFM configurations. More importantly, the coexistence of magnetic order and flat‐band physics is not unique to ${\mathrm{RuOCl}}_{2}$. We have found a series of antiferromagnetic direct‐gapped semiconductors, including ${\mathrm{OsOCl}}_{2}$[32], ${\mathrm{Mn}}_{3}$ ${\mathrm{Sb}}_{2}$ $O_{4}$ $I_{4}$[43], and ${\mathrm{Fe}}_{3}$ ${\mathrm{Sb}}_{2}$ $O_{4}$ ${\mathrm{Br}}_{4}$ [43] (Figure S8), that host unidirectional flat bands. Such an electronic structure ensures that band‐edge optical transitions exhibit strong in‐plane anisotropy, leading to pronounced linear dichroism (LD) and SH effect.

## 3 Conclusion

In summary, we have theoretically revealed a giant LD and magneto‐optical SH effect in 2D antiferromagnets, exemplified by ${\mathrm{RuOCl}}_{2}$, arising from 1D‐flat‐band‐enhanced interband optical transitions. Both the valence and conduction bands exhibit pronounced directional flatness, leading to strongly anisotropic optical absorption along orthogonal crystallographic axes and broadband hyperbolic frequency windows spanning the entire visible range. The material also hosts record‐high LD, reaching 55%, surpassing all previously reported values in 2D systems. The SH and LD effects emerge at distinct photon energies, reflecting a momentum–direction‐dependent crossover between flat and dispersive bands. Both effects persist and are further enhanced in few‐layer films. Our findings establish 2D flat‐band magnets as a rare class of materials where magnetic order and flat‐band physics coexist to produce highly tunable, directionally selective magneto‐optical responses—opening new avenues for next‐generation optical modulators and integrated opto‐spintronic technologies.

## Conflicts of Interest

The authors declare no conflicts of interest.

## Supporting information


**Supporting File**: advs77173‐sup‐0001‐SuppMat.pdf.

## Data Availability

The data that support the findings of this study are available from the corresponding author upon reasonable request.
