# Supplementary material for: Giant Magneto‐Optical Effects in Two‐Dimensional Flat‐Band Antiferromagnets
Source: Adv Sci (Weinh). 2026 Aug 31:e77173. Online ahead of print. doi: 10.1002/advs.77173 (PMC13530369; doi:10.1002/advs.77173)
Supplement: Supplementary file 1 [file ADVS-9999-e77173-s001.pdf]

Supporting Information for
"Giant Magneto-Optical Effects in Two-Dimensional Flat-Band Antiferromagnets"

Ping Yang,[1,2] Wanxiang Feng,[1,*] Siyuan Liu,[1] Shan Guan,[3] Liwei Wen,[4] Wei Jiang,[1,5] Gui-Bin Liu,[1] and Yugui Yao[1,5,†]

[1]*Key Lab of Advanced Optoelectronic Quantum Architecture and Measurement (MOE), Beijing Key Lab of Nanophotonics and Ultrafine Optoelectronic Systems, and School of Physics,Beijing Institute of Technology, Beijing 100081, China*

[2]*Department of Physics, Tianjin Renai College, Tianjin 301636, China*

[3]*State Key Laboratory of Semiconductor Physics and Chip Technologies, Institute of Semiconductors, Chinese Academy of Sciences, Beijing 100083, China*

[4]*College of Science, Henan University of Engineering, Zhengzhou 451191, China*

[5]*International Center for Quantum Materials, Beijing Institute of Technology, Zhuhai, 519000, China*

I. COMPUTATIONAL METHODS

A. First Principles Calculation

The electronic structure calculations were carried out using the projector augmented wave (PAW) method [1], as implemented in the Vienna *ab initio* Simulation Package (VASP) [2]. Exchange-correlation effects were treated using the generalized gradient approximation with the Perdew-Burke-Ernzerhof (GGA-PBE) functional [3]. A plane-wave energy cutoff of 700 eV was used, and the energy convergence criterion was set to 10^{-6} eV. k-point meshes of $16 \times 16 \times 1$ and $8 \times 16 \times 1$ were used to sample the two-dimensional (2D) Brillouin zone for the nonmagnetic and magnetic states of $RuOCl_2$, respectively. Spin-orbit coupling (SOC) was included in all calculations. The on-site Coulomb interaction of the Ru d-orbitals was treated using the GGA+U method [4] with an effective Hubbard U parameter of 3.0 eV. Van der Waals interactions were accounted for using the DFT-D3 method [5]. A vacuum layer of 15 Å was introduced to eliminate interactions between adjacent atomic layers. A $4 \times 3 \times 1$ supercell was constructed for phonon calculations using the PHONOPY package [6]. Once the converged charge density was obtained, maximally localized Wannier functions, including the Ru d-orbitals and the O and Cl p-orbitals, were constructed using the WANNIER90 package [7].

The interlayer binding energy was evaluated as

$$E_b = \left(E_1 + E_{n-1} - E_n\right)/S, \tag{S1}$$

where E_1, E_{n-1}, and E_n are the total energies of the monolayer, $(n-1)$-layer, and n-layer systems, respectively, and S is the in-plane area of the 2D unit cell.

B. Optical Conductivity and Dielectric Function

The AFM $RuOCl_2$ is a semiconductor, and its optical conductivity arise exclusively from interband transitions. These were computed via the Kubo-Greenwood formula [8], as implemented in the WANNIER90 package [7]:

$$\sigma_{\mu\nu}(\omega) = \frac{ie^2\hbar}{N_k V} \sum_{n,m,\boldsymbol{k}} \frac{f_{m\boldsymbol{k}} - f_{n\boldsymbol{k}}}{E_{m\boldsymbol{k}} - E_{n\boldsymbol{k}}} \frac{\langle \psi_{n\boldsymbol{k}} | v_\mu | \psi_{m\boldsymbol{k}} \rangle \langle \psi_{m\boldsymbol{k}} | v_\nu | \psi_{n\boldsymbol{k}} \rangle}{E_{m\boldsymbol{k}} - E_{n\boldsymbol{k}} - (\hbar\omega + i\eta)}, \tag{S2}$$

where N_k is the total number of k-points in the Brillouin zone, η is the energy smearing parameter, $v_{\mu(\nu)}$ denotes the velocity operator along the $\mu(\nu)$ direction, and $\psi_{n\boldsymbol{k}}$ and $E_{n\boldsymbol{k}}$ are the eigenfunction and eigenvalue corresponding to band index n at wavevector $\boldsymbol{k}$, respectively. A dense k-point mesh of $400 \times 400 \times 1$ was employed to ensure convergence of the optical conductivity, and the smearing parameter η was set to 0.1 eV.

Dielectric function contributed by interband transitions is related to the optical conductivity via

$$\varepsilon_{\mu\nu}^{\text{inter}}(\omega) = \delta_{\mu\nu} + \frac{4\pi i}{\omega}\sigma_{\mu\nu}(\omega), \tag{S3}$$

* wxfeng@bit.edu.cn

† ygyao@bit.edu.cn

where $\delta_{\mu\nu}$ is the Kronecker delta, equal to 1 for $\mu = \nu$ and 0 otherwise.

The NM $RuOCl_2$ is a metal (see Figure. S6b), its dielectric function consists of both intraband and interband contributions. Based on the Drude model, the real and imaginary parts of the intraband contribution are given by

$$\mathrm{Re}\left[\varepsilon_{\mu\nu}^{\text{intra}}(\omega)\right] = 1 - \frac{(\omega_{\mu\nu}^{p})^2}{\omega^2+\gamma^2},\ \mathrm{Im}\left[\varepsilon_{\mu\nu}^{\text{intra}}(\omega)\right] \quad = \frac{\gamma(\omega_{\mu\nu}^{p})^2}{\omega(\omega^2+\gamma^2)}, \tag{S4}$$

where ω is the photon frequency, γ is the electron lifetime broadening parameter, set to 50 meV, and $\omega_{\mu\nu}^{p}$ (with $\mu, \nu \in \{x, y, z\}$ denoting Cartesian directions) is the plasma frequency, calculated as [9]

$$\left(\omega_{\mu\nu}^{p}\right)^2 = -\frac{4\pi e^2}{V}\sum_{n,\boldsymbol{k}} 2f'_{n\boldsymbol{k}}\left(\hat{\boldsymbol{e}}_{\mu}\cdot\frac{\partial E_{n\boldsymbol{k}}}{\partial\boldsymbol{k}}\right)\left(\hat{\boldsymbol{e}}_{\nu}\cdot\frac{\partial E_{n\boldsymbol{k}}}{\partial\boldsymbol{k}}\right), \tag{S5}$$

where V is the volume defined as the product of the in-plane area S and the effective thickness d_{eff} (5.633 Å for a monolayer), $\hat{\boldsymbol{e}}_{\mu(\nu)}$ is the unit vector along the $\mu(\nu)$ direction, and $f'_{n\boldsymbol{k}}$ denotes the derivative of the Fermi-Dirac distribution ($f_{n\boldsymbol{k}}$) with respect to the energy eigenvalue $E_{n\boldsymbol{k}}$ for band index n at wavevector $\boldsymbol{k}$. Owing to the highly anisotropic band dispersion, the in-plane plasma frequencies of monolayer $RuOCl_2$ are calculated to be $\omega_{xx}^{p} = 8.16$ eV and $\omega_{yy}^{p} = 0.22$ eV.

C. Dipole Selection Rules

To determine the dipole selection rules, a symmetry analysis was performed using the MagVasp2trace [10, 11] and MSGCorep [12, 13] packages. First, the characters of the wave functions at high-symmetry points and along relevant paths were computed using the MagVasp2trace code. These characters were then processed with the "readMagTrace" command in the MSGCorep package, followed by the "getBandCorep" command to extract the irreducible co-representations at the corresponding points and paths (Figure S4c). It should be noted that the coordinate axes in the 2D slab model of monolayer $RuOCl_2$ (Figure 1a in the main text) do not align with those defined in the MSGCorep package. This discrepancy can be observed from the elements of the magnetic space group P_amma [Figure S4(a)], where the $\frac{1}{2}$ translation operation is along the z-axis in MSGCorep, while it corresponds to the x-axis in our 2D slab model. Consequently, the x, y, and z axes in the 2D model correspond to the z, x, and y axes, respectively, in the MSGCorep setting. As a result, the Γ–X–S–Y path in Figure 1d (main text) corresponds to the Γ–Z–U–X path in Figure S4b, and the k-point labels along this path in different settings are compared in Table S3. Given this axis correspondence, the velocity operators $\hat{v}_x$ and $\hat{v}_y$ are equivalent to the dipole operators $-e\hat{z}$ and $-e\hat{x}$, respectively, which transform as the basis of the irreducible co-representations Γ_4^- and Γ_3^- of the group P_amma. The dipole selection rules were then derived using the "showMSGCorepDirectProduct" command in MSGCorep, which provides the direct products and their decompositions between the irreducible co-representations of dipole operators and wave functions at high-symmetry points. The results of the direct product analysis for the X and S points are shown in Figure S4d and summarized as:

$$\begin{aligned} &\Gamma_4^- \otimes \mathrm{X}_3 = \mathrm{X}_4, \quad \Gamma_4^- \otimes \mathrm{X}_4 = \mathrm{X}_3, \quad \Gamma_3^- \otimes \mathrm{X}_3 = \mathrm{X}_4, \quad \Gamma_3^- \otimes \mathrm{X}_4 = \mathrm{X}_3; \\ &\Gamma_4^- \otimes \mathrm{S}_3 = \mathrm{S}_4, \quad \Gamma_4^- \otimes \mathrm{S}_4 = \mathrm{S}_3, \quad \Gamma_3^- \otimes \mathrm{S}_3 = \mathrm{S}_4, \quad \Gamma_3^- \otimes \mathrm{S}_4 = \mathrm{S}_3. \end{aligned} \tag{S6}$$

These results indicate that the dipole selection rules allow transitions between $X_3 \leftrightarrow X_4$ and $S_3 \leftrightarrow S_4$ for both x- and y-polarized light. For $\mathbf{k}$-points along high-symmetry paths (such as Σ, D, and C), only one irreducible co-representation is present, and thus all interband transitions are symmetry-allowed.

II. SUPPLEMENTAL FIGURES AND TABLES

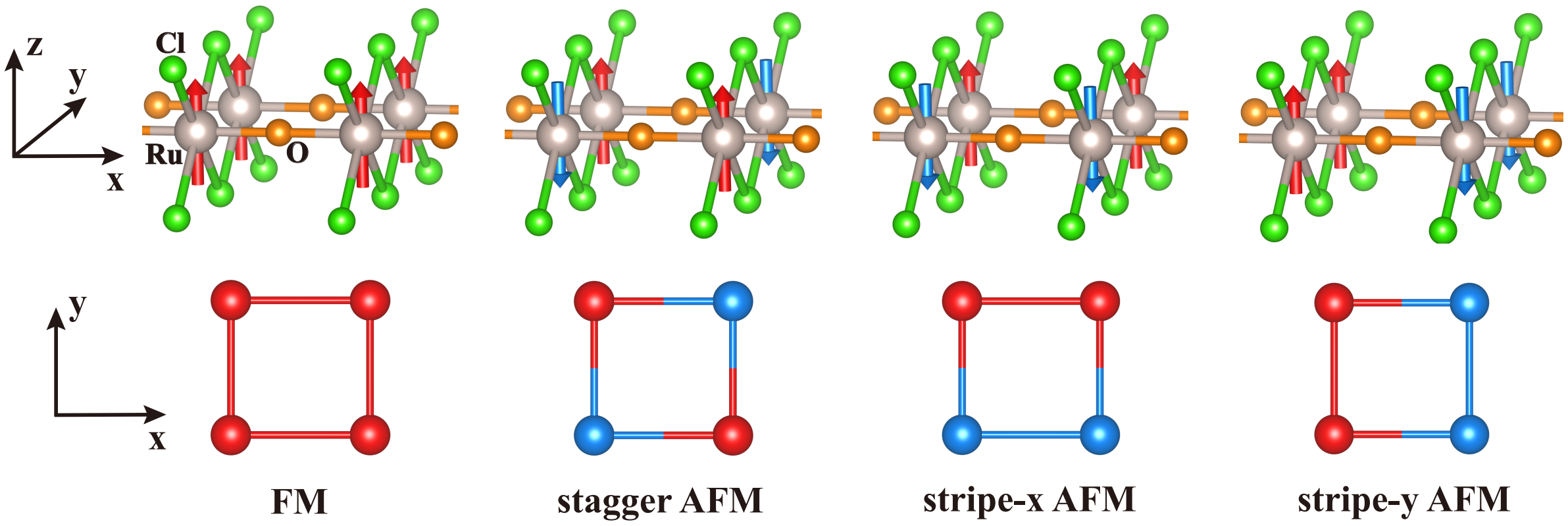

Figure S1. First row: Side views of the crystal and magnetic structures of monolayer $RuOCl_2$ in the ferromagnetic (FM) and three types of antiferromagnetic (AFM) states: stagger, stripe-x, and stripe-y. Second row: Top views of the corresponding magnetic lattices, showing only Ru atoms; nonmagnetic atoms (O and Cl) are omitted for clarity. Red and blue spheres denote Ru atoms with spin-up and spin-down orientations, respectively.

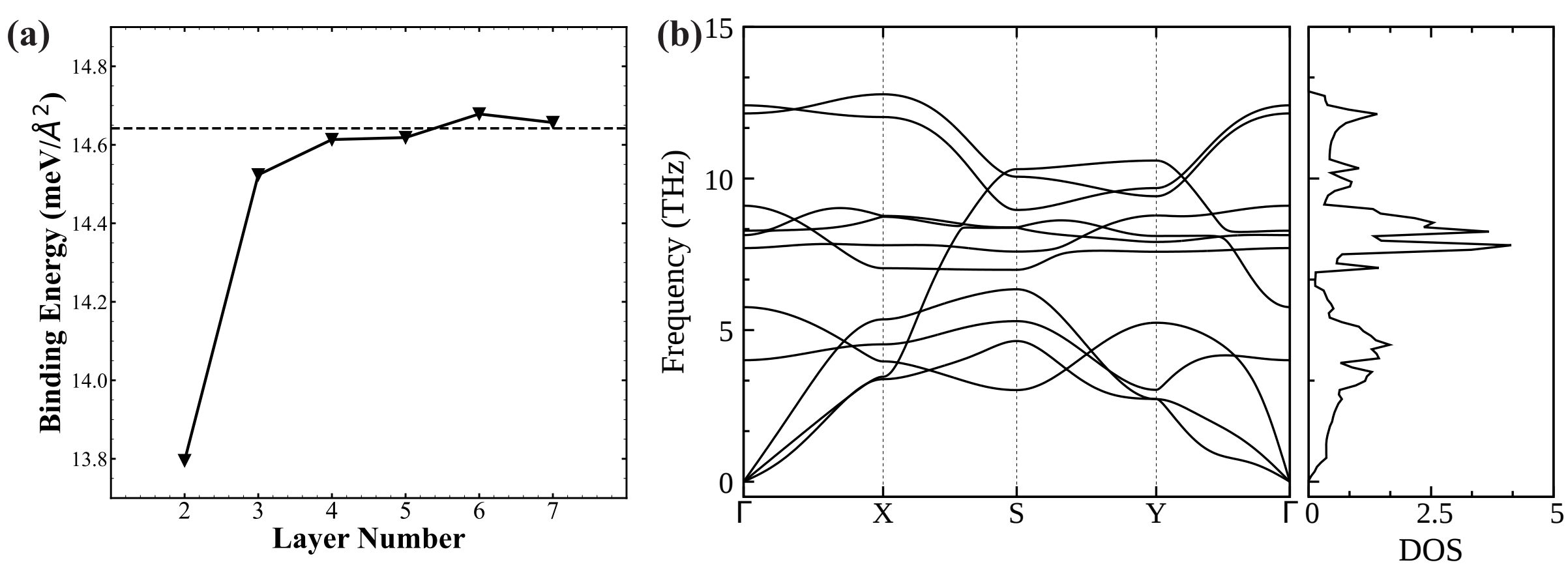

Figure S2. (a) Interlayer binding energy as a function of the number of layers for stripe-y antiferromagnetic (AFM) $RuOCl_2$. The dashed line indicates the converged binding energy of 14.64 meV/Å^2. (b) Phonon spectrum and corresponding phonon density of states for monolayer stripe-y AFM $RuOCl_2$.

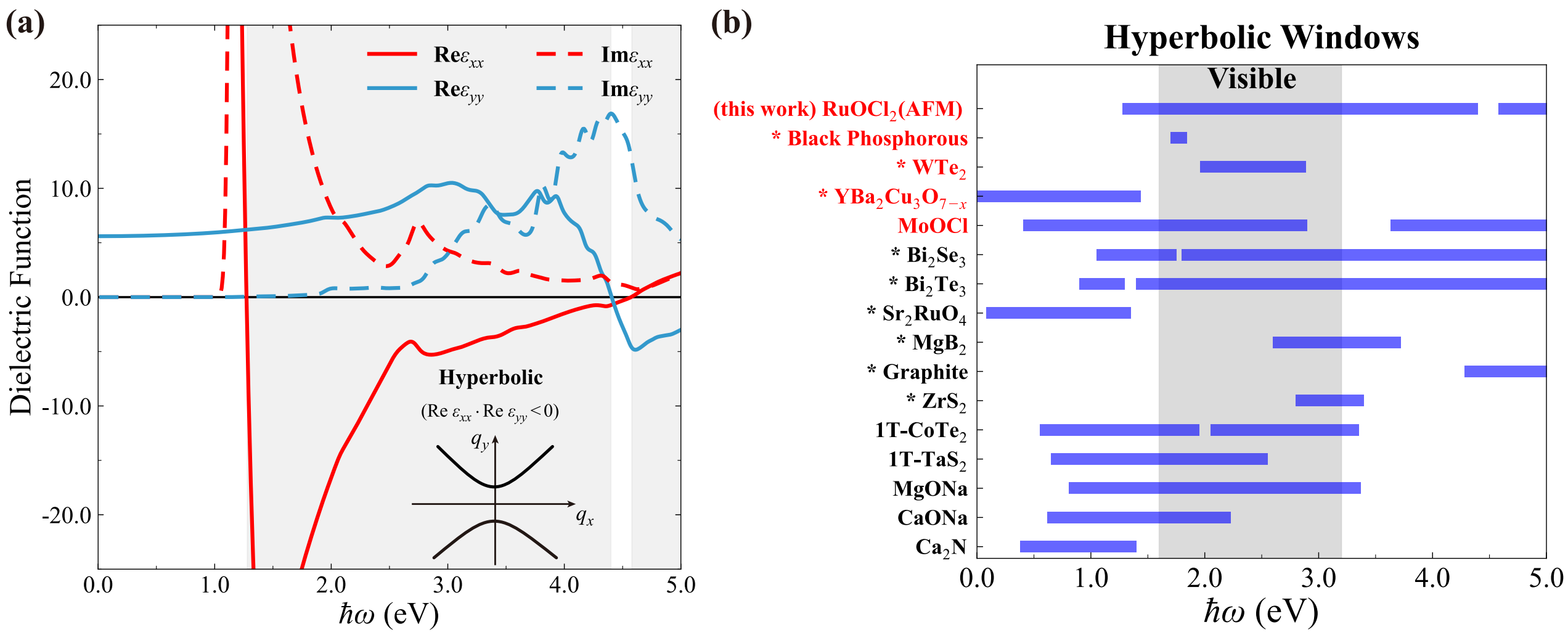

Figure S3. (a) Permittivity of monolayer stripe-y AFM $RuOCl_2$. The inset schematically illustrates the hyperbolic dispersion. Shaded regions indicate hyperbolic frequency windows where $\text{Re}\varepsilon_{xx} \cdot \text{Re}\varepsilon_{yy} < 0$. (b) Hyperbolic windows of monolayer stripe-y antiferromagnetic (AFM) $RuOCl_2$, along with those of other representative 2D materials. The entries marked in red are biaxial crystals with in-plane anisotropy (Black Phospsorus [14], WTe_2 [15],$YBa_2Cu_3O_{7-x}$ [16], $MoOCl_2$ [9]) while the entries in black are uniaxial crystals (Bi_0Se_3 [17], Bi_2Te_3 [17], $SrRuO_3$ [16], MgB_2 [16], Graphite [16], and ZrS_2 [18], 1T-$CoTe_2$ [19], 1T-TaS_2 [19], MgONa [20], CaONa [20], Ca_2N [21]).
The star indicate experimental values, while the unmarked entries represent the results of theoretical calculations. Shaded regions indicate the visible light energy range.

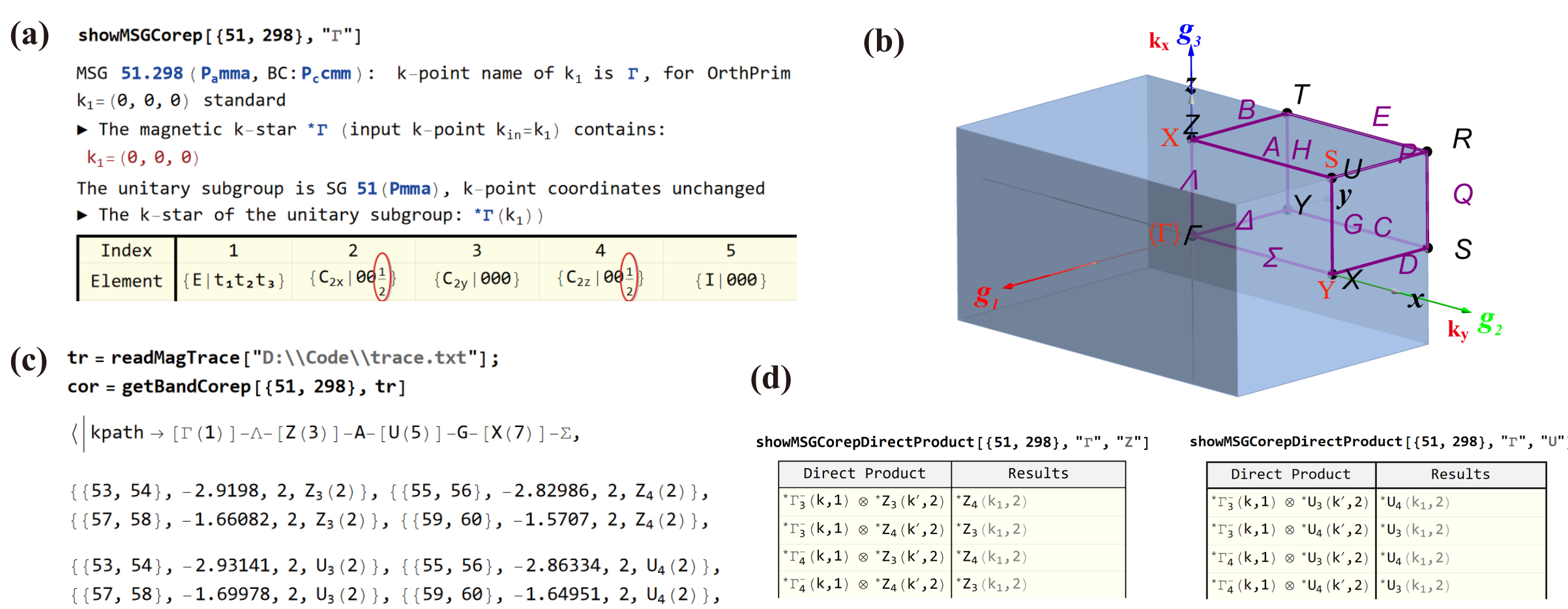

Figure S4. (a) Elements of the magnetic space group P_amma (No. 51.298), generated using the "showMSGCorep" command in the MSGCorep package. The $\frac{1}{2}$ entries highlighted with red circles indicate half-unit-cell translation operations along the z-axis. (b) First Brillouin zone of an orthorhombic crystal, generated using the "showBZDemo" command in MSGCorep. High-symmetry points labeled in black follow MSGCorep conventions, while those in red correspond to the notation adopted in our $RuOCl_2$ 2D slab model. (c) Irreducible co-representations of wave functions at high-symmetry points, obtained using the "getBandCorep" command after reading characters with the "readMagTrace" command in MSGCorep. Band indices 53–56 correspond to the top four valence bands, and 57–60 to the bottom four conduction bands; in both cases, the bands form spin-degenerate pairs as a result of space-time inversion symmetry. (d) Direct products of irreducible co-representations at the Z and U points, corresponding to the X and Y points in our 2D slab model, respectively, generated using the "showMSGCorepDirect-Product" command in MSGCorep.

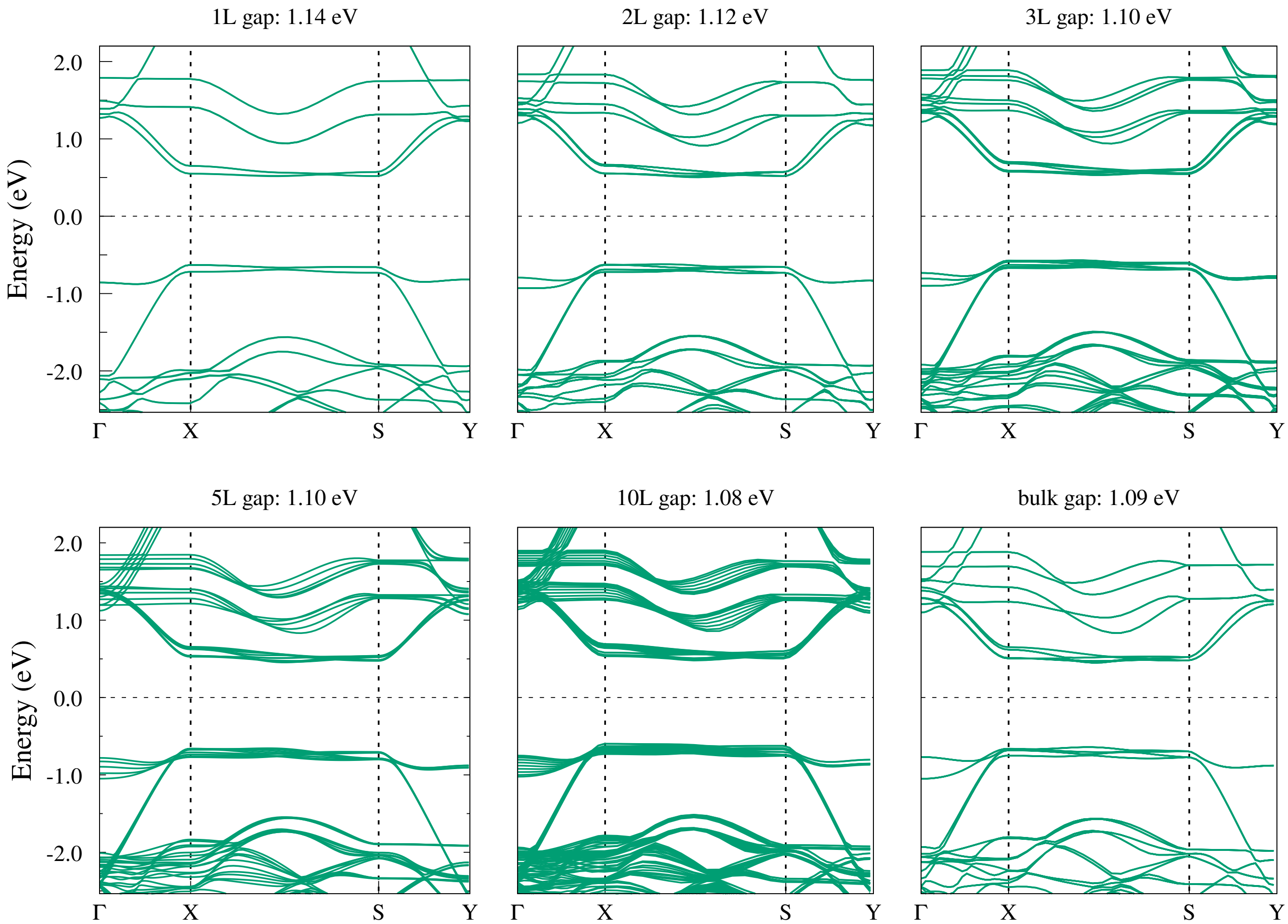

Figure S5. Band structures of stripe-y antiferromagnetic (AFM) $RuOCl_2$ with different thickness.

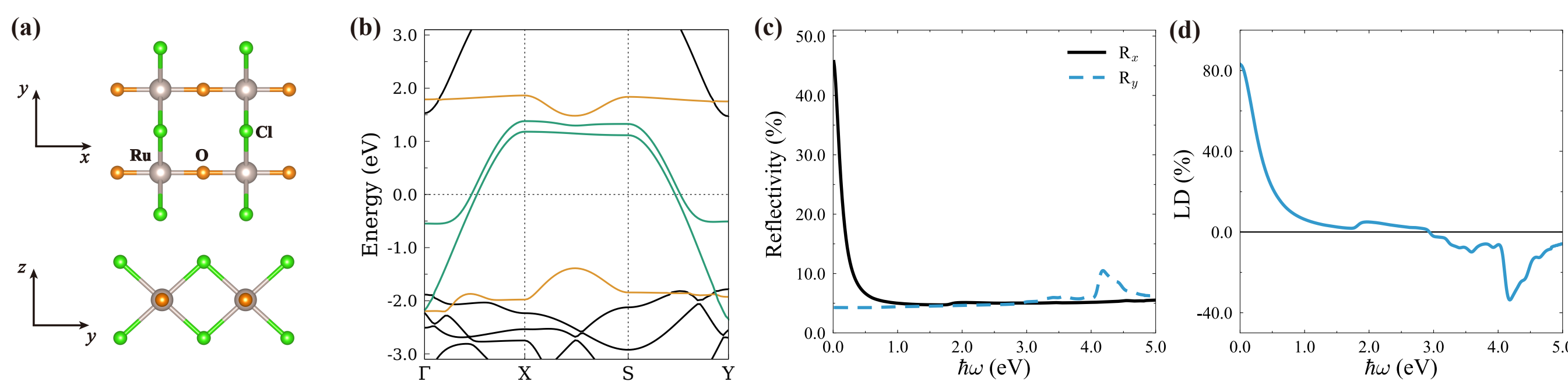

Figure S6. (a) Top and side views of the crystal structure of monolayer nonmagnetic (NM) $RuOCl_2$. (b) Relativistic band structure of monolayer NM $RuOCl_2$. Bands exhibiting nearly flat and strongly dispersive behavior along orthogonal crystallographic directions (Γ–X/S–Y and X–S) are highlighted in green and gold, respectively. (c) Light reflectivity spectra along the x and y directions and (d) linear dichroism spectrum of monolayer NM $RuOCl_2$ on a SiO_2 substrate.

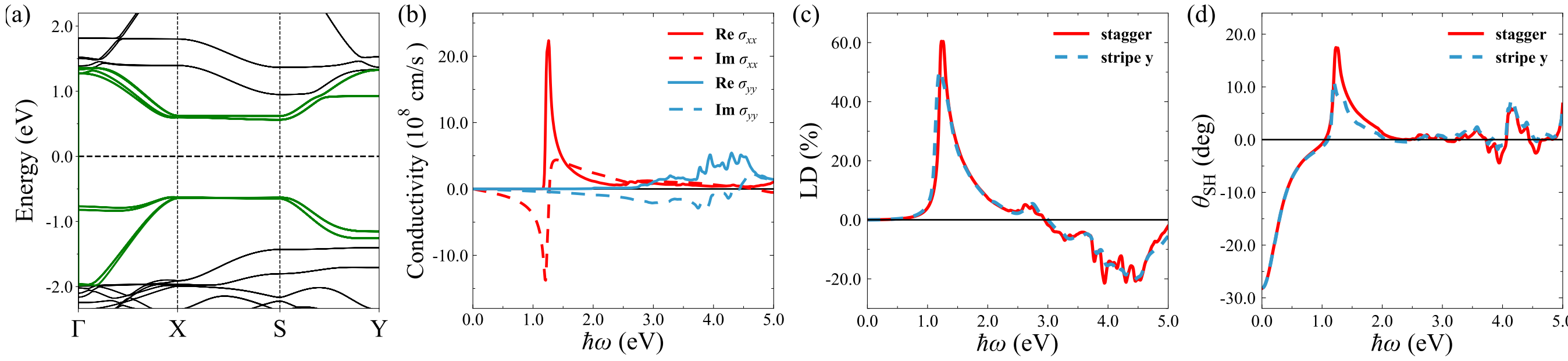

Figure S7. (a) Band structure, (b) optical conductivity, (c) linear dichroism, and (d) Schäfer-Hubert angle of monolayer stagger-AFM $RuOCl_2$. In (c) and (d), the corresponding results for the stripe-y AFM state are shown for comparison.

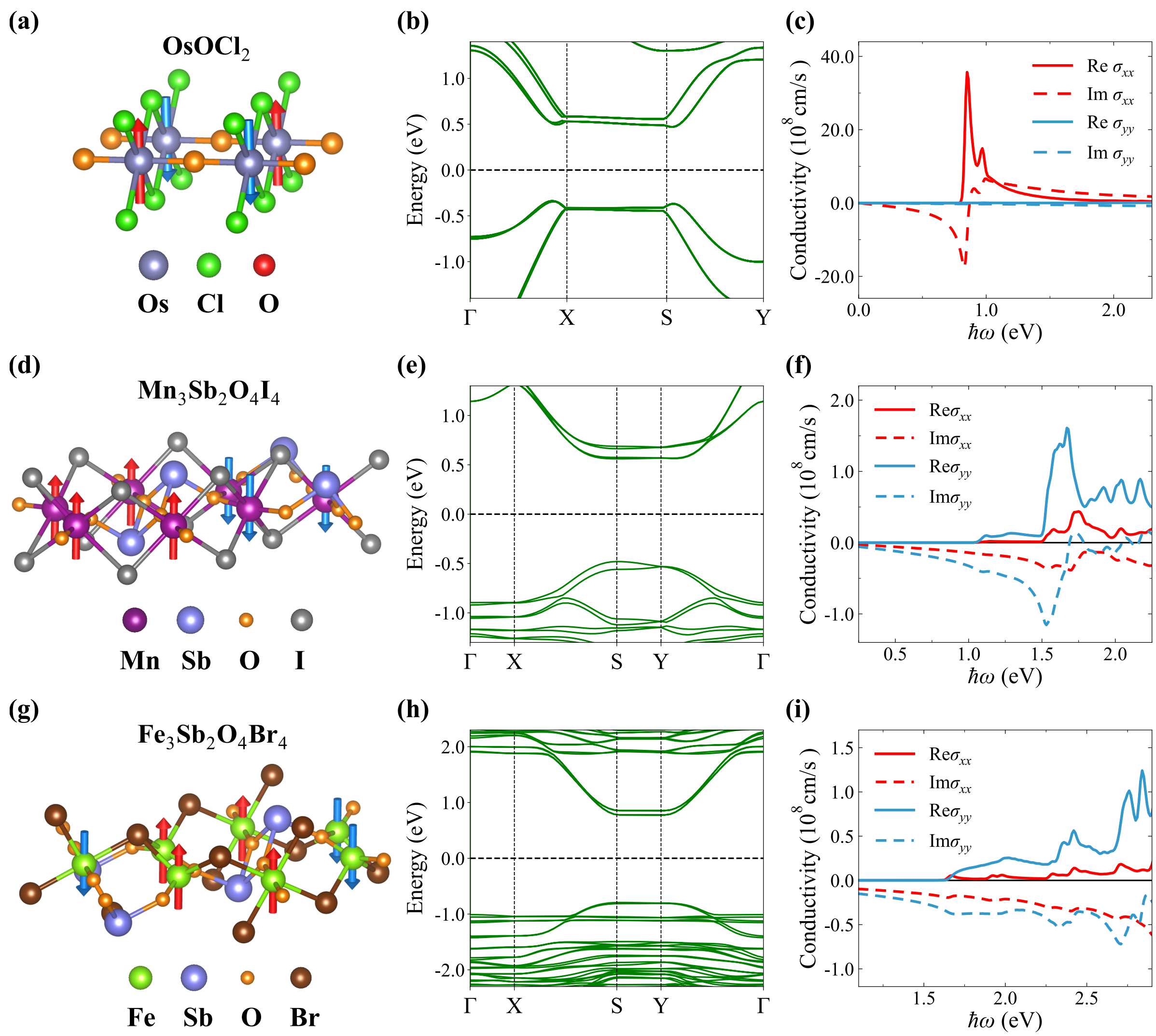

Figure S8. Crystal and magnetic structures (left column), band structures (middle column), and optical conductivities (right column) of monolayer antiferromagnetic $OsOCl_2$, $Mn_3Sb_2O_4I_4$, and $Fe_3Sb_2O_4Br_4$. Strong optical anisotropy is observed in all three materials, originating from the unidirectional flat bands. Pronounced linear dichroism and a large magneto-optical Schäfer-Hubert effect are therefore expected, similar to the case of $RuOCl_2$ discussed in the main text.

TABLE S1. Optimized lattice constants, spin magnetic moments on Ru atoms, and total energies for various magnetic configurations of monolayer $RuOCl_2$ as shown in Figure S1. The listed spin magnetic moments are obtained after electronic relaxation, although the initial values were uniformly set to 3.0 μ_B to represent ferromagnetic (FM) and three types of antiferromagnetic (AFM) configurations. Results for the nonmagnetic (NM) configuration are also included for comparison. The total energy of the stripe-y AFM state is taken as the reference.

Magnetic configuration	Lattice constant a / b (Å)	Magnetic moment (μ_B)	Total energy (meV)
NM	3.645 / 3.526	0.001	190.3
FM	3.645 / 3.526	0.002	190.3
stagger AFM	3.676 / 3.520	0.987	1.82
stripe-x AFM	3.644 / 3.523	0.001	186.5
stripe-y AFM	3.678 / 3.530	0.984	0

TABLE S2. Linear dichroism (LD) and magneto-optical Schäfer-Hubert angle (θ_{SH}) of monolayer stripe-y AFM $RuOCl_2$, in comparison with those of other nonmagnetic (NM), ferromagnetic (FM), and antiferromagnetic (AFM) materials.

Material	Magnetic state	LD	θ_{SH} (mrad)	Wavelength (nm)	Thickness (nm)
$RuOCl_2$ (this work)	NM	83.2 %	491	1.2×10^5 (0.01 eV)	0.563 (1L)
$RuOCl_2$ (this work)	AFM	50.4%	288	1,033 (1.2 eV)	0.563 (1L)
$RuOCl_2$ (this work)	AFM	84.7%	505	1,033 (1.2 eV)	5.633 (10L)
BP [22]	NM	40%		3000	30–50
BP [23]	NM	15%		600	11
ReS_2 [23]	NM	31%		600	62
Nb_3SeI_7 [24]	NM	39%		404	40
CrOCl [25]	NM	30%		600	40
VOCl [26]	NM	7.5%		620	40
GeS [27]	NM	20%		633	172.1
GeS_2 [28]	NM	5%		325	4.2
GeAs [29]	NM	7%		600	20
TlSe [30]	NM	6%		790	18
1T'-WTe_2 [31]	NM	7%		633	16.36
$PdSe_2$ [32]	NM	10%		532	95.8
penta-PdPSe [33]	NM	3%		600	8.44
Violet Phosphorus [34]	NM	3%		700	25
$FePS_3$ [35]	AFM	1.18%	6	633	1.4 (2L)
$NiPS_3$ [35]	AFM	2%	10	633	4 (5L)
$Fe_{1/3}NbS_2$ [36]	AFM		1	633	bulk
Fe_2As [37]	AFM		0.5	783	80
CoO [38]	AFM		0.12	800	10
Mn_3Sn [39]	AFM		0.33	800	40
(Ga,Mn)As [40]	FM		0.85	800	350
CuMnAs [41]	AFM		0.06	920	10
Fe [42]	FM		0.1	633	10
Fe [43]	FM		1.7	X-ray	bulk
NiO [44]	AFM		1	X-ray	20

TABLE S3. Comparison of k-point labels along the high-symmetry path for the 2D slab model adopted in Figure 1d (main text) and in the MSGCorep setting, as also shown in Figure S4b.

2D slab model	Γ	Σ	X	D	S	C	Y
MSGCorep setting	Γ	Λ	Z	A	U	G	X

[1] P. E. Blöchl, *Phys. Rev. B* **1994**, *50*, 24 17953.
[2] G. Kresse, D. Joubert, *Phys. Rev. B* **1999**, *59*, 3 1758.
[3] J. P. Perdew, K. Burke, M. Ernzerhof, *Phys. Rev. Lett.* **1996**, *77*, 18 3865.
[4] S. L. Dudarev, G. A. Botton, S. Y. Savrasov, C. J. Humphreys, A. P. Sutton, *Phys. Rev. B* **1998**, *57*, 3 1505.
[5] S. Grimme, S. Ehrlich, L. Goerigk, *J. Comput. Chem.* **2011**, *32*, 7 1456.
[6] A. Togo, *J. Phys. Soc. Jpn.* **2022**, *92*, 1 012001.
[7] G. Pizzi, V. Vitale, R. Arita, S. Blügel, F. Freimuth, G. Géranton, M. Gibertini, D. Gresch, C. Johnson, T. Koretsune, J. Ibañez-Azpiroz, H. Lee, J.-M. Lihm, D. Marchand, A. Marrazzo, Y. Mokrousov, J. I. Mustafa, Y. Nohara, Y. Nomura, L. Paulatto, S. Poncé, T. Ponweiser, J. Qiao, F. Thöle, S. S. Tsirkin, M. Wierzbowska, N. Marzari, D. Vanderbilt, I. Souza, A. A. Mostofi, J. R. Yates, *J. Phys.: Condens. Matter* **2020**, *32*, 16 165902.
[8] J. R. Yates, X. Wang, D. Vanderbilt, I. Souza, *Phys. Rev. B* **2007**, *75* 195121.
[9] J. Zhao, W. Wu, J. Zhu, Y. Lu, B. Xiang, S. A. Yang, *Phys. Rev. B* **2020**, *102*, 24 245419.
[10] Y. Xu, L. Elcoro, Z.-D. Song, B. J. Wieder, M. G. Vergniory, N. Regnault, Y. Chen, C. Felser, B. A. Bernevig, *Nature* **2020**, *586*, 7831 702.
[11] L. Elcoro, B. J. Wieder, Z. Song, Y. Xu, B. Bradlyn, B. A. Bernevig, *Nat. Commun.* **2021**, *12*, 1 5965.
[12] G.-B. Liu, M. Chu, Z. Zhang, Z.-M. Yu, Y. Yao, *Comput. Phys. Commun.* **2021**, *265* 107993.
[13] G.-B. Liu, Z. Zhang, Z.-M. Yu, Y. Yao, *Comput. Phys. Commun.* **2023**, *288* 108722.
[14] F. Wang, C. Wang, A. Chaves, C. Song, G. Zhang, S. Huang, Y. Lei, Q. Xing, L. Mu, Y. Xie, H. Yan, *Nat. Commun.* **2021**, *12*, 1 5628.
[15] C. Wang, S. Huang, Q. Xing, Y. Xie, C. Song, F. Wang, H. Yan, *Nat. Commun.* **2020**, *11*, 1 1158.
[16] J. Sun, N. M. Litchinitser, J. Zhou, *ACS Photonics* **2014**, *1*, 4 293.
[17] M. Esslinger, R. Vogelgesang, N. Talebi, W. Khunsin, P. Gehring, S. de Zuani, B. Gompf, K. Kern, *ACS Photonics* **2014**, *1*, 12 1285.
[18] S. C. Bayliss, W. Y. Liang, *J. Phys. C* **1982**, *15*, 6 1283.
[19] M. N. Gjerding, R. Petersen, T. G. Pedersen, N. A. Mortensen, K. S. Thygesen, *Nat. Commun.* **2017**, *8*, 1 320.
[20] M.-I. Choe, K.-H. Kim, J.-H. Wi, *ChemPhysChem* **2021**, *22*, 1 92.
[21] S. Guan, S. Y. Huang, Y. Yao, S. A. Yang, *Phys. Rev. B* **2017**, *95*, 16 165436.
[22] H. Yuan, X. Liu, F. Afshinmanesh, W. Li, G. Xu, J. Sun, B. Lian, A. G. Curto, G. Ye, Y. Hikita, Z. Shen, S.-C. Zhang, X. Chen, M. Brongersma, H. Y. Hwang, Y. Cui, *Nat. Nanotechnol.* **2015**, *10*, 8 707.
[23] W. Shen, C. Hu, J. Tao, J. Liu, S. Fan, Y. Wei, C. An, J. Chen, S. Wu, Y. Li, J. Liu, D. Zhang, L. Sun, X. Hu, *Nanoscale* **2018**, *10*, 17 8329.
[24] J.-P. Wang, Y.-Q. Fang, W. He, Q. Liu, J.-R. Fu, X.-Y. Li, Y. Liu, B. Gao, L. Zhen, C.-Y. Xu, F.-Q. Huang, A. J. Meixner, D. Zhang, Y. Li, *Adv. Opt. Mater.* **2023**, *11*, 15 2300031.
[25] T. Zhang, Y. Wang, H. Li, F. Zhong, J. Shi, M. Wu, Z. Sun, W. Shen, B. Wei, W. Hu, X. Liu, L. Huang, C. Hu, Z. Wang, C. Jiang, S. Yang, Q.-m. Zhang, Z. Qu, *ACS Nano* **2019**, *13*, 10 11353.
[26] T. Zhang, J. Du, W. Wang, K. Wu, S. Yue, X. Liu, W. Shen, C. Hu, M. Wu, Z. Qu, S. Yang, C. Jiang, *Nano Res.* **2023**, *16*, 5 7481.
[27] Z. Li, Y. Yang, X. Wang, W. Shi, D.-J. Xue, J.-S. Hu, *ACS Appl. Mater. Interfaces* **2019**, *11*, 27 24247.
[28] Y. Yang, S.-C. Liu, X. Wang, Z. Li, Y. Zhang, G. Zhang, D.-J. Xue, J.-S. Hu, *Adv. Funct. Mater.* **2019**, *29*, 16 1900411.
[29] S. Yang, Y. Yang, M. Wu, C. Hu, W. Shen, Y. Gong, L. Huang, C. Jiang, Y. Zhang, P. M. Ajayan, *Adv. Funct. Mater.* **2018**, *28*, 16 1707379.
[30] S. Yang, C. Hu, M. Wu, W. Shen, S. Tongay, K. Wu, B. Wei, Z. Sun, C. Jiang, L. Huang, Z. Wang, *ACS Nano* **2018**, *12*, 8 8798.
[31] Q. Zhang, R. Zhang, J. Chen, W. Shen, C. An, X. Hu, M. Dong, J. Liu, L. Zhu, *Beilstein J. Nanotechnol.* **2019**, *10* 1745.
[32] L. Pi, C. Hu, W. Shen, L. Li, P. Luo, X. Hu, P. Chen, D. Li, Z. Li, X. Zhou, T. Zhai, *Adv. Funct. Mater.* **2021**, *31*, 3 2006774.
[33] P. Li, J. Zhang, C. Zhu, W. Shen, C. Hu, W. Fu, L. Yan, L. Zhou, L. Zheng, H. Lei, Z. Liu, W. Zhao, P. Gao, P. Yu, G. Yang, *Adv. Mater.* **2021**, *33*, 35 2102541.
[34] W. Chen, A. Chen, R. Zhang, J. Zeng, L. Zhang, M. Gu, C. Wang, M. Huang, Y. Guo, H. Duan, C. Hu, W. Shen, B. Niu, K. Watanabe, T. Taniguchi, J. Zhang, J. Li, X. Cai, G. Liu, *Nano Lett.* **2023**, *23*.
[35] Q. Zhang, K. Hwangbo, C. Wang, Q. Jiang, J.-H. Chu, H. Wen, D. Xiao, X. Xu, *Nano Lett.* **2021**, *21*, 16 6938.
[36] A. Little, C. Lee, C. John, S. Doyle, E. Maniv, N. L. Nair, W. Chen, D. Rees, J. W. F. Venderbos, R. M. Fernandes, J. G. Analytis, J. Orenstein, *Nat. Mater.* **2020**, *19*, 10 1062.
[37] K. Yang, K. Kang, Z. Diao, A. Ramanathan, M. H. Karigerasi, D. P. Shoemaker, A. Schleife, D. G. Cahill, *Phys. Rev. Mater.* **2019**, *3*, 12 124408.
[38] Z. Zheng, J. Y. Shi, Q. Li, T. Gu, H. Xia, L. Q. Shen, F. Jin, H. C. Yuan, Y. Z. Wu, L. Y. Chen, H. B. Zhao, *Phys. Rev. B* **2018**, *98*, 13 134409.
[39] H. C. Zhao, H. Xia, S. Hu, Y. Y. Lv, Z. R. Zhao, J. He, E. Liang, G. Ni, L. Y. Chen, X. P. Qiu, S. M. Zhou, H. B. Zhao, *Nat. Commun.* **2021**, *12*, 1 5266.
[40] A. V. Kimel, G. V. Astakhov, A. Kirilyuk, G. M. Schott, G. Karczewski, W. Ossau, G. Schmidt, L. W. Molenkamp, T. Rasing, *Phys. Rev. Lett.* **2005**, *94*, 22 227203.
[41] V. Saidl, P. Němec, P. Wadley, V. Hills, R. P. Campion, V. Novák, K. W. Edmonds, F. Maccherozzi, S. S. Dhesi, B. L. Gallagher, F. Trojánek, J. Kuneš, J. Železný, P. Malý, T. Jungwirth, *Nat. Photon.* **2017**, *11*, 2 91.
[42] K. Postava, H. Jaffres, A. Schuhl, F. Nguyen Van Dau, M. Goiran, A. R. Fert, *J. Magn. Magn. Mater.* **1997**, *172*, 3 199.
[43] S. Valencia, A. Kleibert, A. Gaupp, J. Rusz, D. Legut, J. Bansmann, W. Gudat, P. M. Oppeneer, *Phys. Rev. Lett.* **2010**, *104*, 18 187401.
[44] J. Xu, C. Zhou, M. Jia, D. Shi, C. Liu, H. Chen, G. Chen, G. Zhang, Y. Liang, J. Li, W. Zhang, Y. Wu, *Phys. Rev. B* **2019**, *100*, 13 134413.
